# Probing the Nanosecond Dynamics of a Designed Three-Stranded Beta-Sheet with a Massively Parallel Molecular Dynamics Simulation

**DOI:** 10.3390/ijms10031013

**Published:** 2009-03-10

**Authors:** Vincent A. Voelz, Edgar Luttmann, Gregory R. Bowman, Vijay S. Pande

**Affiliations:** 1 Department of Chemistry / Stanford Unversity, Stanford, California 94305, USA; E-Mails: vvoelz@stanford.edu (V.V.); luttmann@stanford.edu (E.L.); 2 Biophysics Program / Stanford University, Stanford, California 94305, USA; E-Mail: gbowman@stanford.edu

**Keywords:** Ultrafast folding, downhill folding, DPDP, DPDP-II, designed beta-sheet proteins

## Abstract

Recently a temperature-jump FTIR study of a designed three-stranded sheet showing a fast relaxation time of ~140 ± 20 ns was published. We performed massively parallel molecular dynamics simulations in explicit solvent to probe the structural events involved in this relaxation. While our simulations produce similar relaxation rates, the structural ensemble is broad. We observe the formation of turn structure, but only very weak interaction in the strand regions, which is consistent with the lack of strong backbone-backbone NOEs in previous structural NMR studies. These results suggest that either ^D^P^D^P-II folds at time scales longer than 240 ns, or that ^D^P^D^P-II is not a well-defined three-stranded β-sheet. This work also provides an opportunity to compare the performance of several popular forcefield models against one another.

## 1. Introduction

Xu *et al.* have recently studied the nanosecond time scale folding dynamics of a designed three-stranded sheet mini-protein [1]. This peptide, called ^D^P^D^P-II, is one of many peptide sequences originally designed by the Gellman group for the purposes of elucidating the sources of thermodynamic stability and folding cooperativity of beta-hairpin and beta-sheet structures [2]. The most stable of these beta-sheet designs have a scaffold that incorporates successive D-proline and glycine residues (^D^PG) in the turn regions, a motif shown to form a stable Type-II′ turn [3].

**Figure 1.**
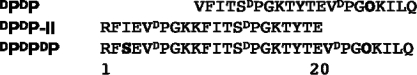
Designed beta-sheet peptides designed by the Gellman group: ^D^P^D^P [4], ^D^P^D^P-II [5], and ^D^P^D^P^D^P [5]. ^D^P denotes D-Proline, and O denotes Ornithine.

Of great interest as model systems have been several three- and four-stranded beta-sheet designs from the Gellman group. The first of these, ^D^P^D^P, was studied by NMR and shown to have cross-strand NOEs and chemical shifts indicative of beta-sheet populations [4]. Syud *et al.* built upon ^D^P^D^P, producing (among others): ^D^P^D^P-II, a three-stranded sheet whose C-terminal hairpin is identical to the N-terminal hairpin of ^D^P^D^P (Figure 1); and ^D^P^D^P^D^P, a four-stranded composite of ^D^P^D^P and ^D^P^D^P-II [5]. The stability of these designs was assessed in a similar fashion by NMR.

Recently, designed ^D^PG-turn beta-sheet peptides have become interesting candidates for ultrafast folding beta-sheet systems. Many proteins have been engineered to fold quickly [6–8], close to the “speed limit” of folding [9,10]. Upper limits on protein folding rates are thought ultimately to be controlled by the conformational search rate for forming intermolecular contacts [11,12]. For beta-sheet proteins, the entropic barriers of turn formation are rate limiting [13]. Indeed, a designed variant of human Pinl WW domain, with a ^D^PG substitution in the turn region, shows a 10-fold increase in folding rate compared to native sequence, up to (~10 μs)^−1^, becoming one of the fastest folding beta-sheet proteins to date [13].

It had been hypothesized that because of the reduced conformational entropy of the ^D^PG turn regions, the folding landscape of ^D^P^D^P-II might not have activation barriers to folding, and shown to be that of a “downhill” folder [8], with kinetics shaped mainly by landscape roughness. Using temperature-jump FTIR, Xu *et al.* showed that ^D^P^D^P-II has “the fastest T-jump relaxation rate observed for a beta-sheet system so far” of (~140 ± 20 ns)^−1^, with single-exponential relaxation kinetics [1]. More recently, T-jump FTIR studies of the related four-stranded peptide, ^D^P^D^P^D^P, show similar single-exponential kinetics, but with a folding time of ~440 ns [14].

While the single-exponential kinetics of ^D^P^D^P-II can be fit to a two-state Arrhenius-type model, Xu *et al.* showed that one-dimensional Langevin models of dynamics over a rough free-energy surface [15,16] explain the data equally well, which is their preferred interpretation. In the case of the four-stranded ^D^P^D^P^D^P, Xu *et al.* suspect that many parallel but degenerate refolding pathways may be present [14].

One reason to prefer the “downhill” interpretation is the lack of features typical of activated folding kinetics [15,16]. The fast rate of the relaxation (on the time scale of helix-coil transitions) imply that unfolded and folded ensembles must have a similar degree of compactness, and end-to-end distances as measured by FRET show little sensitivity to temperature. Xu *et al.* suggest a reason for this is the reduced accessible conformational space imposed by the rigid ^D^PG turns. Smith and Tokmakoff used time-resolved infrared spectroscopy along with site-specific isotopic labeling techniques to show that the ^D^PG turn region of de novo hairpin peptide PG12 does not undergo significant rearrangement upon a temperature-jump, where as the mid-strand regions rearrange on a ~130-ns time scale [17]. Their results support a model where the unfolded state is an expanded but native-like ensemble.

Simulation studies have shed some light onto the thermodynamics of ^D^PG-turn beta-sheet proteins. While there have been no previous simulations of ^D^P^D^P-II, several groups have simulated the related ^D^P^D^P peptide, which shares an 11-residue stretch of hairpin residues (Figure 1). Wang and Sung simulated a 100 ns molecular dynamics trajectory of ^D^P^D^P using an implicit solvent model, starting from an extended conformation [18]. Their results show ^D^P^D^P folding to beta-sheet structures, and agree with experimental findings that the ^D^PG turn is more stable than designed three-stranded peptides with NG or GS turns. Roe *et al.* used replica exchange molecular dynamics in a modified AMBER99 forcefield with an implicit solvation model to sample the thermodynamics of ^D^P^D^P [19]. REMD (12 replica trajectories each of ∼130 ns) dramatically enhanced the convergence of the free energy landscape compared to single-replica MD. The two hairpins of ^D^P^D^P show simulated populations of ~50% and ~75%, respectively, consistent with NMR and CD studies [4,20], and estimates of thermodynamic cooperativity of −1 to −3 kcal/mol. The less stable of the two ^D^P^D^P hairpins comprises the C-terminal sequence of ^D^P^D^P-II.

We have been interested in ^D^P^D^P-II as a target for molecular simulation for several reasons. It appears to be the fastest-folding beta-sheet protein so far, with relaxation kinetics within the time scale range that can be effectively addressed with all-atom molecular simulation. Moreover, the experimental kinetics remain ambiguous as to whether activation barriers exist for this peptide. To investigate the underlying conformational dynamics, we perform massively parallel molecular dynamics simulations of ^D^P^D^P-II in explicit solvent.

As we report below, the reaction coordinates of average radius of gyration and solvent-accessible surface area of backbone C=O over time show good agreement with the experimentally measured relaxation rates, but we observe very few three-stranded sheet structures folded within 240 ns, regardless of the forcefield model used. These results suggest that either ^D^P^D^P-II folds at time scales longer than 240 ns, or that ^D^P^D^P-II is not a stable well-defined β-sheet, which is consistent with previous NMR spectroscopic data [5].

## 2. Results and Discussion

**Figure 2.**
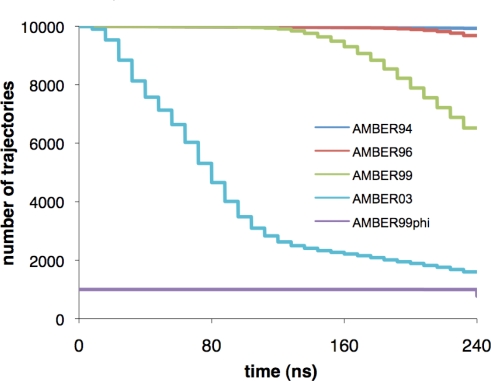
The distribution of trajectories achieving a given trajectory length, shown for the forcefields tested in this study.

The Folding@Home distributed computing platform [21] was used to simulate molecular dynamics (MD) trajectories, each up to 240 ns in length, for five different forcefields, for a total of ~8.2 ms of simulation. Simulations were performed using the GROMACS simulation package [22], with AMBER94 [23], AMBER96 [24], AMBER99 [25], AMBER99ϕ [26], and AMBER03 [27] forcefields (see Methods section). 1000 total trajectories were generated for the AMBER99ϕ simulations, and 10,000 trajectories each were generated for the other forcefield simulations. Figure 2 shows the distribution of trajectory lengths for each forcefield tested.

### 2.1. Simulated relaxation kinetics

Time-resolved FTIR measurements cannot directly determine whether a protein is folded, but instead report the status of backbone amide groups, which may be closely related. To best connect with the relaxation rates experimentally measured using FTIR [1], we therefore analyzed the ensemble time course of the total solvent-accessible surface area of backbone C=O groups, as well as the average radius of gyration of the entire molecule. In general, reaction coordinates must be carefully chosen because a poor choice of can yield projection-dependent results [28,29]. The C=O solvent-accessible surface area is a measure that closely connects with the measured amide I band, which is known to be sensitive to hydration status [30]. The radius of gyration is a global quantity that does a good job of characterizing the structural distribution and compactness of a conformational ensemble.

**Figure 3.**
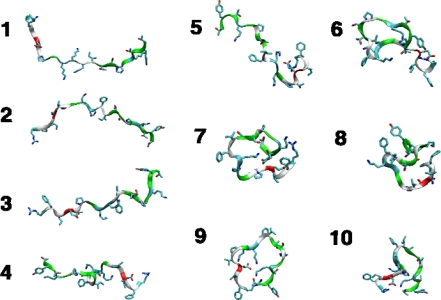
Ten different starting conformations taken from a high-temperature trajectory were used to seed the simulations.

**Figure 4.**
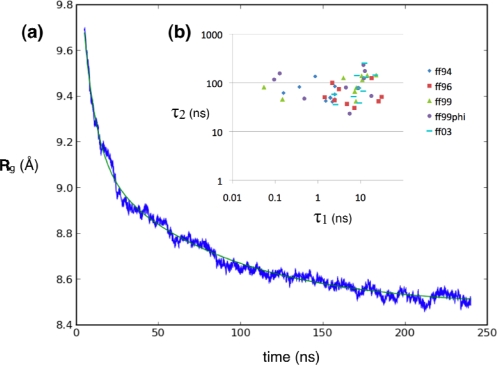
Simulated relaxation kinetics for ^D^P^D^P-II, as characterized by the average radius of gyration, (a) An example trace of the average radius of gyration over time (blue) with the best-fit bi-exponential curve (green), (b) Fitted bi-exponential time constants τ_1_ and τ_2_ across all forcefields and starting conformations.

We simulated 1,000 trajectories each (100 each for AMBER99ϕ) from 10 different starting configurations (Figure 3) taken randomly from a high-temperature equilibration trajectory of ^D^P^D^P-II started from a semi-extended state (see Methods). Figure 4a shows a typical trace of the ensemble-average radius of gyration over time (for a particular combination of forcefield and starting conformation), which fits well to a bi-exponential curve. Figure 6a shows a typical trace of the ensemble-average C=O solvent-accessible surface area over time. The kinetics also fit well to a bi-exponential curve. In both cases, the kinetics show a fast equilibration phase (usually τ_1_ ~1–10 ns) and a slower relaxation phase (τ_2_ ~100 ns). Similar kinetics were computed across all forcefields and starting conformations (Figure 4b). In most cases, the fast phase corresponds to fast equilibration of the starting conformation. Alternatively, in some cases, the fitted values of τ_1_ were extremely short (~0.1 ns), less than the snapshot frequency, indicating that the kinetics may be better described as a single-exponential process with rate constant τ_2_. Numerical values for all fitted kinetic parameters are shown in the Supplementary Material (Tables S1 and S2).

**Figure 5.**
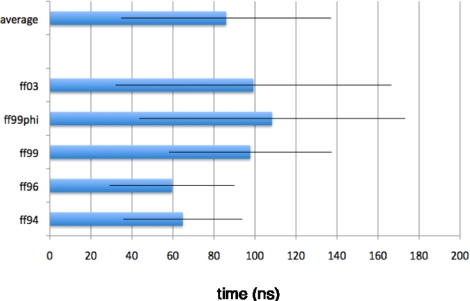
Average simulated relaxation times for ^D^P^D^P-II, for each forcefield, as characterized by average radius of gyration. Error estimates are computed from the standard deviation across the 10 starting conformations.

**Figure 6.**
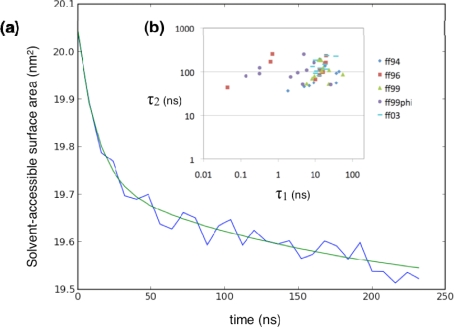
Simulated relaxation kinetics for ^D^P^D^P-II, as characterized by average C=O solvent-accessible surface area. Description is as Figure 4.

**Figure 7.**
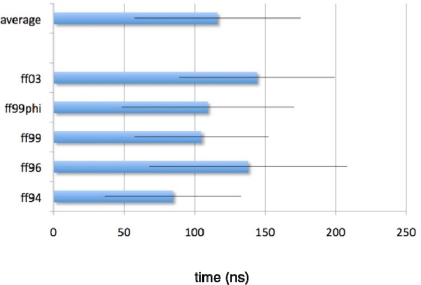
Average simulated relaxation times for ^D^P^D^P-II, for each forcefield, as characterized by average C=O solvent-accessible surface area. Error estimates are computed from the standard deviation across the 10 starting conformations.

Regardless of forcefield choice, the slow relaxation times estimated from our simulations are consistent, ranging from about ~60 to ~100 ns for the radius of gyration reaction coordinate (Figures 4b, 5), and ~80 to ~150 ns (Figures 6b, 7) for the solvent-accessible surface area. Both compare very favorably to the experimentally measured relaxation time of ~140 ± 20 ns obtained by T-jump infrared spectroscopy [1].

The agreement between simulated and experimental rates is comparable to other contemporary examples of physical kinetics simulations [31]. The slightly faster relaxation rates observed in the simulations may in part reflect the anomalously high diffusion constant of the TIP3P water model [32].

The average radius of gyration at 240 ns across all forcefields is 8.32Å ± 0.47Å, and the average value of the exponential baseline, *C*, is 8.28Å ± 0.47Å. This reflects a more conformationally expanded ensemble than seen in simulations of ^D^P^D^P, which showed radius of gyration of ~7Å for unfolded states, ~6.5Å for partially unfolded states, and ~5.5Å for a fully strand-paired native-state conformation [19].

### 2.2. Secondary structures over time

The per-residue secondary structure over time for each forcefield was calculated using the DSSP algorithm [33]. The general features observed across the different forcefields include fast formation of the ^D^PG turn regions, and negligible amounts of sheet formation as quantified by the amount of backbone hydrogen-bonded strand content (see Supplementary Material). It should be noted that strand content may be somewhat underestimated due to the stringent definition required by DSSP.

The amounts of secondary structure across different forcefields reproduce previously noted secondary structural biases [26]. For example, AMBER94 is slightly biased toward more helical conformations and has more populated turn regions (as defined by DSSP), while AMBER96 biased toward beta-sheet conformations, which detectable populations of strand (see Supplementary Material). The more modern forcefields of AMBER99, AMBER99phi, and AMBER03 all show comparable amounts of secondary structural propensities intermediate between AMBER94 and AMBER96. In all cases the DSSP populations are relatively static after ~100 ns.

### 2.3. Hairpin formation over time

#### 2.3.1. QH1 and QH2 at 100–150 ns and 200–230 ns overtime

**Figure 8.**
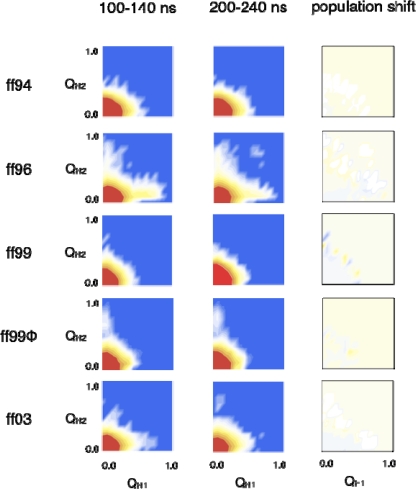
Conformational landscapes for ^D^P^D^P-II. Histograms of sampled populations were constructed in reaction coordinates Q_H1_ and Q_H2_, which monitor the fraction of hairpin 1 and hairpin 2 contacts, respectively. Populations at times 100–140 ns and 200–240 ns are shown in the first two columns. The third column shows a difference map of the population shift over this time. Distributions are plotted on a log-scale, with each color gradation representing one unit k_B_T of free energy at room temperature.

To examine hairpin formation, we computed two quantities, Q_H1_ and Q_H2_, reporting the fraction of “native” contacts in (N-terminal) hairpin 1 and (C-terminal) hairpin 2, respectively (see Methods). The quantities Q_H1_ and Q_H2_ were used as reaction coordinates to compute the landscape of sampled conformations at two time slices: 100–140 ns and 200–240 ns (Figure 8).

Regardless of the choice of forcefield, the conformational landscape mostly disfavors the formation hairpin. Recall that the less stable of the two ^D^P^D^P hairpins comprises the C-terminal sequence of ^D^P^D^P-II, corresponding to hairpin 2. With the exception of the AMBER96 simulations, only the formation of hairpin 2 is mostly observed, and only then with a population of 3% or less at 200 ns. For the AMBER96 trajectories, formation of both hairpin 1 and hairpin 2 is observed. For all the simulations, comparisons of the conformational landscape at 100 ns and 200 ns shows very little change in hairpin populations on the ~100 ns time scale (Figure 8).

**Figure 9.**
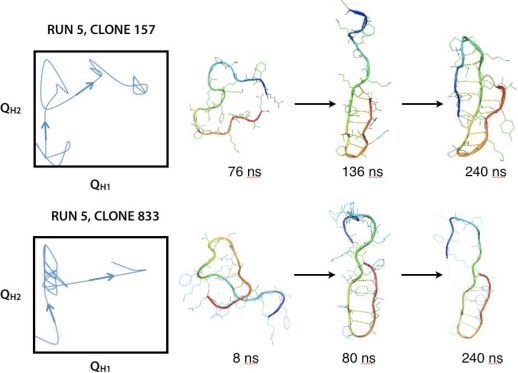
Only two of 10,000 AMBER96 trajectories show folding events for ^D^P^D^P-II within 240 ns. Shown is the time course of reaction coordinates Q_H1_ and Q_H2_, which monitor the fraction of hairpin 1 and hairpin 2, with conformational snapshots. The second of the two trajectories is “native” by our reaction-coordinate definition, although hairpin 1 does not have a fully hydrogen-bonded structure.

Folding to a three-stranded sheet is observed for only two out of a total of 10,000 AMBER96 trajectories (Figure 9). One of these two trajectories shows a fully hydrogen-bonded three-stranded sheet structure, while the other shows only hairpin 2 with defined hydrogen bonds, but is otherwise “native” according to inter-residue contacts defined by the Q_H1_ and Q_H2_ reaction coordinates.

Across all of the forcefields we studied, most all of our simulations do not produce stable three-standed hairpin conformations. We think that this result is very unlikely to be due to poor sampling. With as many as 10,000 simulation replicas per forcefield, there should be a strong likelihood of observing at least some trajectories reaching the folded state [34]. It is possible that forcefield deficiencies may be at work here, but we tested a wide range forcefields, and consistently found negligible amounts of three-stranded. Parallel simulation techniques to accelerate kinetic sampling also has its limits on short timescales where first-passage times are short compared to the folding time [35], but that is not the case here. If the experimentally observed relaxation does indeed correspond to folding, then the overlap in simulated and experimental relaxation time scales should be very favorable for observing transitions to native conformational ensembles.

Is ^D^P^D^P-II a stable folded three-stranded sheet? While Syud *et al.* reported qualitative NOE data for ^D^P^D^P-II, this peptide was the least well-folded compared to the other designed sequences in this paper [5]. The measured NMR resonances were weak, and aggravated by poor dispersion, so only key inter-residue contacts hinting at the designed structure were reported (Syud and Gellman, personal communication). Combined with our simulation results, this suggests that perhaps ^D^P^D^P-II is unstable as a three-stranded sheet, and may not be a very relevant model system for studying beta sheet peptides.

Similar plasticity has observed in another designed three-stranded sheet, the betanova peptide [36]. Both betanova and the ^D^PG-turn peptides of Gellman *et al.* were designed with stable turns and hydrogen-bonded strand regions, to be used as model systems to study beta-sheet cooperativity. WW domains, by contrast, are three-stranded beta-sheet proteins found in nature, whose structures are well-defined [37]. Unlike designed beta-sheet peptides, WW domains additionally possess a conserved network of hydrophobic interactions between their termini. Thus, in general, beta-sheet model systems such as ^D^P^D^P-II may not have the necessary amount of long-range cooperative interactions needed to fully stabilize their structure.

### 2.4. Conformational clustering and Markov State Model (MSM) analysis

Kinetics-based conformational clustering was performed for all snapshots from the AMBER96 trajectories. The AMBER96 trajectories were chosen as they contained the greatest extent of beta-sheet structure, and the only observed folding events. Our clustering procedure was used to identify five macrostate clusters calculated to be the most metastable, which were used to construct Markov State Models [38–40] of the dynamics (see Methods).

We constructed a series of MSMs from matrices of macrostate transition counts, using different lag times ranging from 8 to 240 ns. The performance of these models reveals much about the underlying folding landscape.

**Figure 10.**
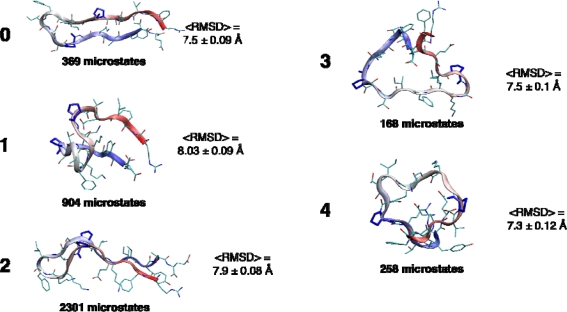
Kinetics-based clustering was used to find five maximally metastable macrostates (see Methods). The representative conformations shown for each state are the most probable conformations in that state. Shown next to each representative conformation is the average RMSD between microstates in that cluster, a measure of the compactness of the conformational ensemble, and the number of microstates (of 4000 total) comprising each macrostate.

The most striking result of the MSM-building procedure was our failure to identify well-separated metastable states that would indicate large activation barriers on the folding landscape. The first indication of this comes from our clustering algorithm, designed to identify the most kinetically metastable states. Only five metastable states were identified, and each contained a broad ensemble of microstate conformations, with average RMSD between any two microstates ranging from 7.3–8.0 Å (Figure 10).

Regardless of lag time, the spectrum of relaxation rates predicted by the MSM is broad, without a large gap that would indicate a pronounced separation of time scales (Figure S2, Supplementary Material). These results, at least within the time scale of our simulations, are not inconsistent with either multistate folding or the “downhill” folding interpretation of Xu *et al.* Moreover, as we increase the lag time used to build the models, the longest implied timescale also increases. If clear activation barriers were present, such that metastable dynamics on the > 10 ns time scale resulted, the implied timescales should level off as the lag time increases. This result is not simply a consequence of poor state definitions, because the kinetic clustering procedure we use should insure that the macrostates are the most metastable basins.

The variability of simulated relaxations across the ten starting conformations offer an additional indication of the absence of large barriers. This is not only evident from the bi-exponential fits of average radius of gyration and average solvent accessible surface area over time, but also from individual MSMs we built using trajectory data generated from each conformation (Figures S3 and S4, Supplementary Material). Similar kinds of heterogeneity in relaxation dynamics for different starting conformations have been observed in previous parallel simulations of ultrafast folders [9].

**Figure 11.**
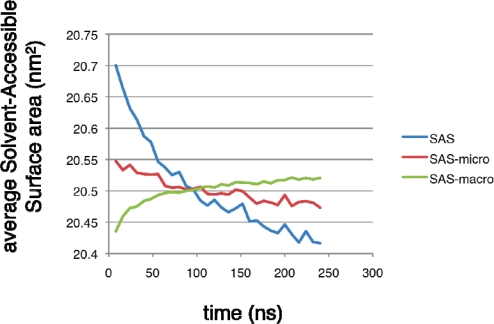
Average C=0 solvent-accessible surface area (SAS) over time computed from simulation snapshot data (blue), compared to the average SAS of each microstate projected onto the 4000 microstate populations over time (red), and average SAS of each macrostate projected onto 5 macrostate populations over time (green). The differential effects produced by averaging over microstates and macrostates indicate a sensitive dependence of the SAS observable on short-lived expanded conformations.

The other striking result of our MSM-building procedure is the unexpected sensitivity of the average C=O solvent-accessible surface area (SAS) to expanded states (Figure 11). When we use the average SAS of each macrostate to compute a projection of the time evolution of the SAS observable, the effects of averaging over each macrostate is severe enough to produce a signal that increases over time instead of decreasing. When the average SAS is projected onto each microstate, this effect is less severe, yet is still present. The simulation data suggest that the average SAS is more sensitively dependent on expanded conformations that quickly collapse, as compared to more compact conformations. Given our good overall results in recapitulating experimentally observed relaxation rates, we remain confident that our representative set of starting conformations is a useful ensemble to compare with FTIR T-jump experiments. However, the SAS projections underscore the importance of choosing experimental observables that overlap well with the reaction coordinate of interest (in this case, the folding reaction) as to best report the underlying dynamics.

One question partially addressed by our work is how experiment and simulation might be used to distinguish between two-state vs. “downhill” folding. As we have shown, one potential indication of so-called “downhill” folding from simulations might be a failure to build a Markovian kinetic model able to describe dynamics as transitions between well-defined metastable states. However, our work suggests that perhaps ^D^P^D^P-II is not a well-defined beta-sheet structure, which brings into question what is meant by “folding” in this case.

Can simulations help suggest experiments that could discriminate downhill vs. activated folding? This is a challenging task, as the observed experimental kinetics for “downhill” folders may depend on many factors. Liu and Gruebele, using one-dimensional Langevin models, present an excellent elucidation of the possible experimental outcomes that can arise from slight differences in folding landscapes (such as native-biases, roughness, and barrier heights) and the reaction coordinate-dependence of reporter probes [16]. Using simulations to identify observables that connect well with folding reaction-coordinates may be particularly useful. For example, our simulations of ^D^P^D^P-II suggest that the C=O solvent-accessible surface area (SAS) is more sensitive to expanded versus compact states. The insensitivity may be in part because the SAS is an aggregate measure across all peptide residues. To the extent that the SAS correlates with the amide I band spectroscopic observable in FTIR T-jump experiments, we suggest that multiple time-resolved FTIR experiments using isotopic labeling of specific residues, combined with microscopic information about peptide conformations from simulation, would help to better resolve folding landscapes for ultrafast folding proteins.

## 3. Experimental Section

### 3.1. System preparation and simulation protocol

Ten initial starting conformations were selected iteratively from a 1 ns stochastic dynamics (SD) simulation at 3000K, with 9 Å cutoffs for Coulomb and vdW interactions, integration time step of 1 fs, neighbor searching on a grid every 10 steps, at solvent (shear) viscosity of 10 ps^−1^. Ten conformations were picked iteratively from a collection of snapshots saved every 1 ps. After picking the first conformation, the most diverse structure (as measured by RMSD) was picked as the next. This procedure was repeated to create a structurally diverse starting set. Each chosen (nearly random) structure was then minimized and equilibrated for the production runs.

Production runs were performed using the TIP3P water model [32] for explicit solvation. A rhombic dodecahedral box of largest dimension 58.7Å was used with periodic boundary conditions. The box contained a ^D^P^D^P-II molecule with uncapped termini, approximately 4,650 water molecules (this number varied slightly with starting conformation) and two chloride counterions to achieve a net neutral charge. Molecular dynamics (MD) simulations were ran at 308 K in the NVT ensemble with a 2 fs integration time step. The same cut-off and neighbor-list settings above were employed, along with a reaction-field electrostatics model, Berendsen temperature coupling, and constrained bonds with the LINCS algorithm. Trajectory snapshots were recorded every 100 ps. Total C=O solvent-accessible surface area was calculated for each snapshot from the set of all carbon and oxygen atoms in the backbone carbonyl groups, using a solvent probe radius of 1.4Å.

### 3.2. Exponential curve fitting

Best-fit parameters β*=(*A, B, C,* τ_1_, τ_2_) for bi-exponential curves of the form *f*(*t*) = *A*exp(−t/τ_1_) + *B*exp(−t/τ_2_) *+ C* were calculated for time series of the average radius of gyration and C=O solvent-accessible surface area, by using a simulated annealing protocol to minimize the sum of squared errors. The first 5 ns of the time series were omitted from the fitting procedure. Variances $\sigma_{i}^{2}$ in average radius of gyration at each time point *i* were calculated by non-parametric bootstrap of 100 samples. Errors in parameter estimates for each *β_j_* were calculated as diagonal elements of the covariance matrix **C**(β^*^) *=* (**F**^T^**WF**)^−1^, where **F** is the (N × 5) Jacobian matrix

(2) $$F_{ij}\;=\;{\frac{\partial f(t_{i},\;\beta )}{\partial \beta_{j}}|}_{\beta^{*}}$$

and **W** is an N x N diagonal matrix of inverse variances: $W_{ij}\;=\;1/\sigma_{i}^{2}$ for *i*=*j*, *W_ij_* = 0 for *i*≠*j* [41].

### 3.3. Secondary structure and “native” hairpin contacts

The DSSP algorithm was used to assess the extent of helix, strand (sheet), turn, and loop secondary structures [33]. DSSP recognizes eight types of secondary structures based on hydrogen bonding patterns: G (3_10_ helix), H (alpha helix), I (pi helix), B (beta bridge), E (extended sheet), T (turn), S (loop). We monitor helix content as the total of G, H, I, the strand content as the total of B and E.

Q_H1_ and Q_H2_ report the fraction of native contacts present for (N-terminal) hairpin 1 and (C-terminal) hairpin 2, respectively. We use the same criteria derived by Roe *et al.,* who used a model of the native conformation to define “native” contacts in each of the two possible hairpins [19]. For hairpin 1, the set of native sidechain contacts (C_α_ for glycine) is defined as residue pairs (R1,I3), (R1,T12), (F2,11), (I3,V5), (I3,T12), (E4,G7), (E4,K9), (V5,F10) and native backbone hydrogen bonds (Rl-H, T12-O), (Rl-O, T12-H), (I3-H, F10-O), (I3-O, F10-H). For hairpin 2, the set of native sidechain contacts is defined as residue pairs (K8,F10), (K8,20), (K9,20), (F10,T17), (F10,T19), (I11,S13), (I11,Y18), (I11,E20), (T12, DP-14), (T12,G15), (T12,T17), (S13,Y8) and native backbone hydrogen bonds (K9-H, E20-O), (I11-H, Y18-0), (II1-0, Y18-H), (S13-H, K16-0). A contact between sidechains is defined when centroid distances < 6.5Å and a backbone contact is defined when hydrogen donor-acceptor distance < 2.5Å.

### 3.3. Kinetics-based clustering for building Markov State Models (MSM)

Representative conformations were extracted from the simulation data using a procedure previously described [42], though constant temperature simulations were used. This method uses Markov State Models (MSMs) to identity kinetically related regions of phase space. Thus, two conformations will be found in the same state if a simulation can move between them quickly but will be grouped into different states if transitioning between them is slow. The definitions of fast and slow are based on the timescales observed in the simulations [39,42].

The first step in building such an MSM is to group conformations with a high degree of structural similarity into small sets called microstates. In this study 4,000 microstates were generated based on their all-atom RMSD using a *k*-centers clustering algorithm [43]. A desirable feature of this algorithm is that the resulting microstates have approximately equal volumes so their populations are directly related to their densities, or free energies. If each microstate is sufficiently small then it is assumed that structural similarity is equivalent to kinetic similarity since it should take a very short time to transition between very similar conformations. Kinetically related microstates, as judged by the number of transitions between them observed in the data, are then grouped together using the PCCA algorithm and this lumping is refined using a simulated annealing scheme [38,44,45]. The center of the most populated microstate from each macrostate is then selected as the representative conformation for that macro state as it is the most probable.

### 3.4. Markov State Model (MSM) construction

The matrix of transition probabilities **T** between the five macrostates was computed from the trajectory data. The entries of this matrix *T_ij_* contain the probability of transitioning from state *i* to state *j* in time τ, which ranged from 8 ns to 240 ns. Diagonalization of (**T**^T^ − **1**) produces a set of eigenvalues *μ_k_* and corresponding eigenvectors **e**_k_ which describe the dynamics of state populations **p**(*t*) as a linear combination of relaxation processes:

(1) $$\text{p}(t)\;=\;\sum_{k}\alpha_{i}{\text{e}}_{k}e^{-\lambda_{k}t}$$

where λ_k_=[ln *μ_k_*]/τ, and the α*_i_* are determined by the initial state populations **p**(0) [38,39]. Thus λ_k_^−1^ are the set of implied timescales involved in the relaxation dynamics.

## 4. Conclusions

We performed massively parallel folding simulations of ^D^P^D^P-II to investigate the conformational dynamics underlying its nanosecond refolding dynamics. The simulated relaxation rates, as monitored by average radius of gyration and average C=O solvent-accessible surface area, agree well with the single-exponential relaxation rates experimentally measured by T-jump FTIR. Furthermore, Markov state models built from the trajectory data do not show a separation of metastable timescales consistent with large activation barriers. These results, at least within the time scale of our simulations, are not inconsistent with either multistate folding or the “downhill” folding interpretation of Xu *et al.* However, despite the agreement with experimental kinetics, we observe very few trajectories that fold to stable three-stranded beta-sheet structures. These results suggest that either ^D^P^D^P-II folds at time scales longer than 240 ns, or that ^D^P^D^P-II is not a well-defined three-stranded β-sheet. The latter interpretation is consistent with previous NMR spectroscopic data [5].

## Supplementary Materials

Supplementary materials are available online at http://www.mdpi.eom/1422-0067/10/3/1013/sl.

## Supplementary Material

Figure S1. Secondary structure profiles overtime, for all forcefields tested, as classified by the DSSP algorithm of Kabsch and Sander (1983). Consistent across all forcefields is the rapid formation of the ^D^PG turns, but negligible amounts of strand formation. The simulations under different forcefields also reproduce long-known secondary structural biases; for example, the helical propensity of AMBER94 compared to more modern forcefields.

Figure S2. (a) A MSM built using a lag time of τ=240 ns reproduces the time evolution of macrostate populations, (b) As the lag time used to build the MSM increases, so do the implied timescales (shown with error bars from a simple bootstrapping procedure). Regardless of lag time, the implied timescales do not show a pronounced separation of timescales.

Figure S3. Markov State Models built from trajectory data for each starting conformation, each constructed using a short lag time of τ=8 ns. MSM predictions of the macrostate population time evolution is shown as the solid line; the actual macrostate populations over time are shown as dots.

Figure S4. Implied timescales as a function of lag time for MSMs constructed for each starting conformation. Error estimates (bars) for timescales at each lag time were derived from a bootstrapping procedure.

Figure S5. Bi-exponential fits of the average C=O solvent-accessible surface area (SAS) over time computed from simulation snapshot data (blue), compared to the average SAS of each microstate projected onto the 4000 microstate populations over time (red), and average SAS of each macrostate projected onto 5 macrostate populations over time (green). Despite the differential effects produced by averaging over microstates and macrostates, the relaxation time scales are similar.

**Table S1.** Parameters for bi-exponential curves *A* exp(t/τ_1_) + *B* exp(t/τ_2_) + *C* fitted to the average radius of gyration over time for each forcefield model and starting conformation.

model	conf	A (Å)		B(Å)		C(Å)		τ _1_(ns)		τ _2_ (ns)	
**ff94**	**0**	5.033	± 0.345	0.794	± 0.004	7.795	± 0.001	2.493	± 0.360	**57.757**	±0.354
	**l**	5.006	0.704	0.517	0.004	7.820	0.001	1.967	0.473	**48.438**	0.259
	**2**	4.950	0.374	0.461	0.003	8.147	0.002	2.507	0.389	**84.182**	0.751
	**3**	5.018	0.397	0.846	0.005	8.133	0.001	2.506	0.426	**45.982**	0.345
	**4**	4.981	0.744	1.107	0.007	8.240	0.001	2.187	0.623	**40.717**	0.362
	**5**	5.070	41.314	0.561	0.003	7.656	0.004	0.867	5.824	**134.93**	1.281
	**6**	5.014	-	1.404	0.004	7.948	0.001	0.157	-	**61.837**	0.449
	**7**	4.952	-	0.460	0.002	7.678	0.001	0.370	-	**81.967**	0.448
	**8**	5.032	0.693	0.663	0.004	7.864	0.001	1.953	0.460	**49.997**	0.282
	**9**	5.020	1.677	0.444	0.004	8.241	0.001	1.533	0.717	**42.272**	0.256

**ff96**	**0**	5.043	0.662	0.992	0.009	9.350	0.002	2.454	0.688	**43.309**	0.563
	**1**	4.955	0.587	0.566	0.008	8.929	0.002	2.456	0.606	**43.875**	0.476
	**2**	4.979	4.446	0.500	0.006	9.163	0.002	1.442	1.670	**50.432**	0.543
	**3**	4.837	0.832	0.239	0.004	9.379	0.005	2.177	0.668	**99.665**	1.574
	**4**	4.807	0.364	1.074	0.007	9.697	0.004	3.115	0.583	**74.105**	1.387
	**5**	−1.262	1.172	1.304	1.176	9.037	0.004	26.411	7.628	**41.577**	11.188
	**6**	−1.433	0.108	1.515	0.019	8.750	0.001	4.805	0.474	**36.572**	0.632
	**7**	−1.541	0.962	1.045	0.964	8.997	0.005	31.123	7.958	**51.380**	13.122
	**8**	0.550	0.016	0.400	0.010	8.444	0.012	18.133	0.522	**124.259**	5.210
	**9**	0.522	0.028	0.404	0.030	8.628	0.001	7.164	0.402	**30.423**	0.610

**ff99**	**0**	1.113	0.027	0.779	0.006	7.840	0.002	7.309	0.227	**66.321**	0.655
	**1**	0.963	0.014	0.332	0.004	7.921	0.008	10.618	0.227	**138.224**	2.921
	**2**	0.771	0.016	0.561	0.005	8.095	0.007	11.026	0.286	**114.416**	2.380
	**3**	1.067	0.026	0.672	0.007	8.477	0.003	7.963	0.268	**79.218**	1.161
	**4**	1.440	0.014	0.604	0.006	8.258	0.013	14.344	0.403	**141.921**	5.159
	**5**	1.009	-	0.612	0.002	7.829	0.002	0.055	-	**81.050**	0.513
	**6**	1.096	-	1.305	0.004	8.079	0.001	0.149	-	**45.977**	0.327
	**7**	−1.639	0.077	0.604	0.003	7.716	0.005	3.980	0.195	**125.616**	1.470
	**8**	1.128	0.022	0.494	0.014	7.866	0.001	7.816	0.279	**41.492**	0.485
	**9**	0.533	0.021	0.089	0.010	8.270	0.017	23.216	0.601	**143.168**	7.718

**ff99ϕ**	**0**	1.020	0.028	0.630	0.021	8.386	0.035	8.861	0.622	**77.890**	14.298
	**1**	1.160	0.029	0.437	0.017	7.850	0.030	12.616	0.599	**174.122**	11.908
	**2**	0.708	0.138	0.639	0.154	8.289	0.006	17.907	2.385	**53.769**	4.931
	**3**	1.388	0.240	0.942	0.013	8.387	0.008	4.552	0.780	**80.028**	2.651
	**4**	1.479	0.199	1.406	0.114	8.655	0.003	5.569	1.561	**23.287**	1.543
	**5**	0.358	-	0.872	0.006	7.841	0.008	0.095	-	**116.315**	2.487
	**6**	0.587	0.049	1.159	0.056	7.737	0.073	11.707	0.863	**232.291**	31.895
	**7**	0.497	-	0.437	0.012	7.852	0.015	0.128	-	**155.409**	4.771
	**8**	1.197	0.031	0.332	0.009	7.963	0.016	12.026	0.645	**123.404**	5.806
	**9**	0.545	-	0.624	0.008	8.160	0.003	0.482	-	**47.257**	0.713

**ff03**	**0**	1.285	0.027	0.630	0.011	8.386	0.006	8.861	0.381	**77.890**	2.070
	**1**	0.931	0.024	0.533	0.009	7.943	0.014	8.203	0.255	**140.234**	4.637
	**2**	0.583	0.015	0.218	0.008	8.442	0.018	13.182	0.433	**128.283**	6.417
	**3**	0.382	0.016	0.825	0.057	8.213	0.068	12.315	0.392	**251.316**	29.238
	**4**	1.795	0.049	0.839	0.018	8.550	0.004	6.876	0.450	**52.116**	1.267
	**5**	0.657	0.356	0.361	0.005	8.190	0.003	2.423	0.369	**58.054**	0.680
	**6**	0.834	0.044	0.664	0.019	8.273	0.042	22.166	1.213	**143.512**	17.958
	**7**	−0.681	0.026	0.352	0.033	8.085	0.002	9.314	0.451	**38.338**	1.003
	**8**	0.525	0.019	0.833	0.020	8.202	0.005	11.543	0.452	**67.408**	1.962
	**9**	0.448	0.275	0.571	0.008	8.487	0.002	2.592	0.343	**35.544**	0.396

Blank error estimates represent cases where the fitted τ_1_ was very close to zero, which our error model treats poorly (see Methods).

**Table S2.** Parameters for bi-exponential curves *A* exp(t/τ_1_) + *B* exp(t/τ_2_) + *C* fitted to the average solvent-accessible surface area of backbone C=0 atoms over time for each forcefield model and starting conformation.

model	conf	A (nm ^2^)			**B (nm ^2^)**		C (nm ^2^)		τ _1_(ns)			τ _2_ (ns)	
**ff94**	**0**	0.314		±0.06	0.263	±0.101	19.46	± 0.147	13.20		±1.22	191.22	±57.71
	**1**	0.127		0.06	0.422	0.054	19.50	0.008	1.85		1.44	36.41	2.56
	**2**	0.135		1.64	0.242	1.525	19.56	0.135	36.17		25.43	92.36	105.64
	**3**	0.127		0.09	0.281	0.084	19.22	0.015	9.12		1.18	55.98	5.99
	**4**	0.220		0.12	0.382	0.051	19.90	0.105	18.18		2.49	135.26	42.12
	**5**	−0.133		0.10	−0.093	0.073	19.78	0.033	12.73		1.72	84.80	12.77
	**6**	0.496		2.15	0.078	1.999	19.75	0.162	42.56		34.48	101.07	140.83
	**7**	0.149		9.51	−0.145	9.481	19.33	0.047	35.93		72.40	55.47	125.59
	**8**	0.078		0.09	0.253	0.076	19.42	0.012	6.75		0.90	47.88	4.59
	**9**	0.135		0.08	0.218	0.064	19.52	0.011	4.96		0.81	45.57	3.79

**ff96**	**0**	0.112		0.10	0.638	0.084	20.46	0.025	10.10		1.41	67.55	9.27
	**1**	0.270		0.10	0.541	0.054	20.28	0.069	13.07		1.82	110.48	25.30
	**2**	0.265		0.14	0.442	0.075	20.16	0.179	18.05		3.11	155.05	71.44
	**3**	0.268		0.15	−0.314	0.082	20.56	0.201	19.13		3.39	160.73	82.07
	**4**	0.032		0.03	0.756	0.078	20.81	0.095	0.62	-		168.33	30.95
	**5**	−0.408		0.16	0.139	0.108	20.54	0.066	16.06		2.90	98.79	26.95
	**6**	−0.203		0.06	0.301	0.048	20.71	0.010	0.04	-		43.51	3.10
	**7**	−0.199		0.14	−0.451	0.103	20.29	0.047	12.97		2.28	84.86	18.38
	**8**	0.412		0.12	0.215	0.286	19.91	0.399	19.35		3.04	233.22	178.51
	**9**	0.043		0.05	0.523	0.221	19.91	0.242	0.70	-		258.40	98.31

**ff99**	**0**	0.153		0.07	0.423	0.057	19.90	0.014	5.79		0.74	53.42	4.59
	**1**	0.311		0.08	0.404	0.059	19.72	0.032	9.57		1.26	79.88	11.15
	**2**	0.272		0.09	0.340	0.067	19.79	0.027	8.08		1.18	70.72	9.35
	**3**	0.187		0.06	0.577	0.098	19.64	0.144	12.41		1.21	185.05	55.65
	**4**	0.355		0.08	0.482	0.111	19.97	0.172	14.40		1.62	183.55	67.44
	**5**	−0.301		1.18	0.330	1.162	19.83	0.028	22.91		12.15	52.47	29.19
	**6**	0.306		15.03	0.421	14.842	20.03	0.204	53.59		174.55	87.42	398.29
	**7**	0.084		0.29	0.065	0.205	19.58	0.102	23.27		5.61	109.14	47.79
	**8**	0.262		0.11	0.173	0.068	19.89	0.061	14.83		2.11	105.48	23.74
	**9**	0.309		0.13	0.445	0.062	19.82	0.093	16.30		2.56	121.68	36.46

**ff99ϕ**	**0**	0.691		0.39	0.444	0.008	19.92	0.010	3.35		0.72	95.62	3.01
	**1**	1.018		0.21	0.758	0.022	19.80	0.005	4.52		0.78	51.41	1.65
	**2**	1.599		0.99	−0.574	0.997	20.00	0.012	25.66		9.87	50.49	18.24
	**3**	0.601		1.39	0.681	0.009	19.85	0.007	2.18		1.14	76.76	1.99
	**4**	0.985		0.13	0.461	0.011	20.23	0.016	5.78		0.67	108.88	5.16
	**5**	0.522	-		0.188	0.009	19.80	0.008	0.14	-		80.45	2.28
	**6**	0.520	-		0.919	0.011	19.99	0.014	0.32	-		123.03	4.24
	**7**	0.511	-		0.196	0.008	19.65	0.009	0.32	-		90.98	2.56
	**8**	0.567		0.19	0.339	0.068	19.91	0.076	4.86		0.67	255.40	31.96
	**9**	0.587		0.06	0.520	0.025	19.91	0.040	9.23		0.80	160.88	14.86

**ffO3**	**0**	0.402		0.11	0.372	0.056	19.74	0.134	18.01		2.42	147.19	52.60
	**1**	0.413		0.06	0.435	0.112	19.53	0.153	10.09		1.04	181.95	55.88
	**2**	0.279		0.14	0.579	0.345	19.35	0.474	20.10		3.35	237.59	212.92
	**3**	0.134		0.09	0.294	0.044	19.50	0.080	13.43		1.60	120.90	28.45
	**4**	0.159		0.07	0.653	0.047	19.97	0.042	10.26		1.19	92.81	14.30
	**5**	−0.121		0.06	−0.047	0.047	19.77	0.079	8.39		1.04	131.58	26.29
	**6**	0.169		0.45	0.626	0.346	19.70	0.775	35.17		11.06	230.31	399.18
	**7**	−0.127		0.20	−0.154	0.114	19.54	0.108	19.30		3.82	114.75	44.76
	**8**	0.163		0.12	0.313	0.091	19.55	0.039	12.76		1.95	81.74	15.11
	**9**	0.141		0.08	−0.012	0.048	19.48	0.057	10.94		1.45	103.82	19.78

Blank error estimates represent cases where the fitted τ_1_ was very close to zero, which our error model treats poorly (see Methods).
