# Supplementary figures and images for: Probing the Nanosecond Dynamics of a Designed Three-Stranded Beta-Sheet with a Massively Parallel Molecular Dynamics Simulation
Source: Int J Mol Sci. 2009 Mar 10;10(3):1013–30. doi: 10.3390/ijms10031013 (PMC2672016; doi:10.3390/ijms10031013)

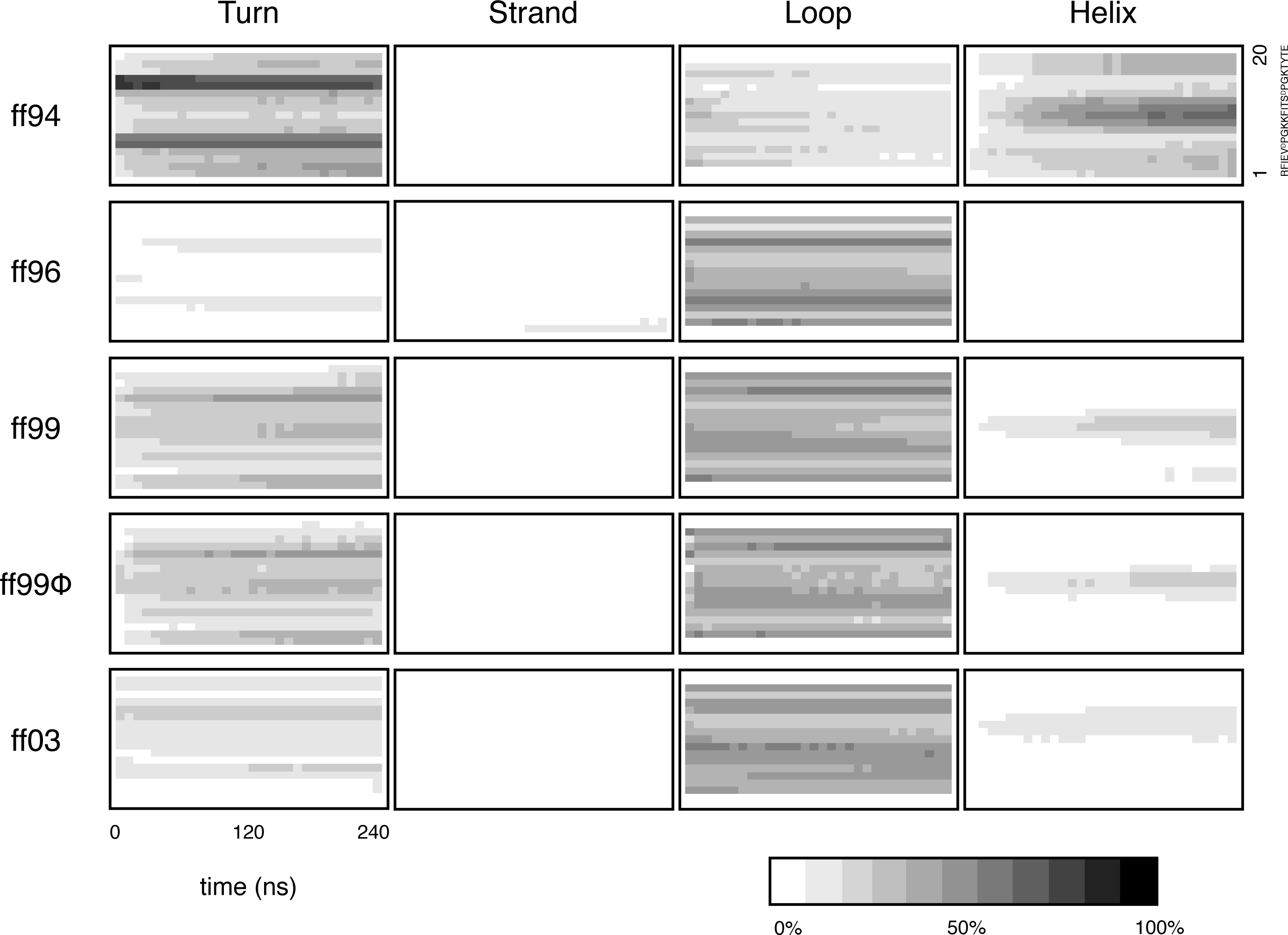

Supplement: Figure S1. — Secondary structure profiles overtime, for all forcefields tested, as classified by the DSSP algorithm of Kabsch and Sander (1983). Consistent across all forcefields is the rapid formation of the DPG turns, but negligible amounts of strand formation. The simulations under different forcefields also reproduce long-known secondary structural biases; for example, the helical propensity of AMBER94 compared to more modern forcefields. [file ijms-10-01013f12.tif]

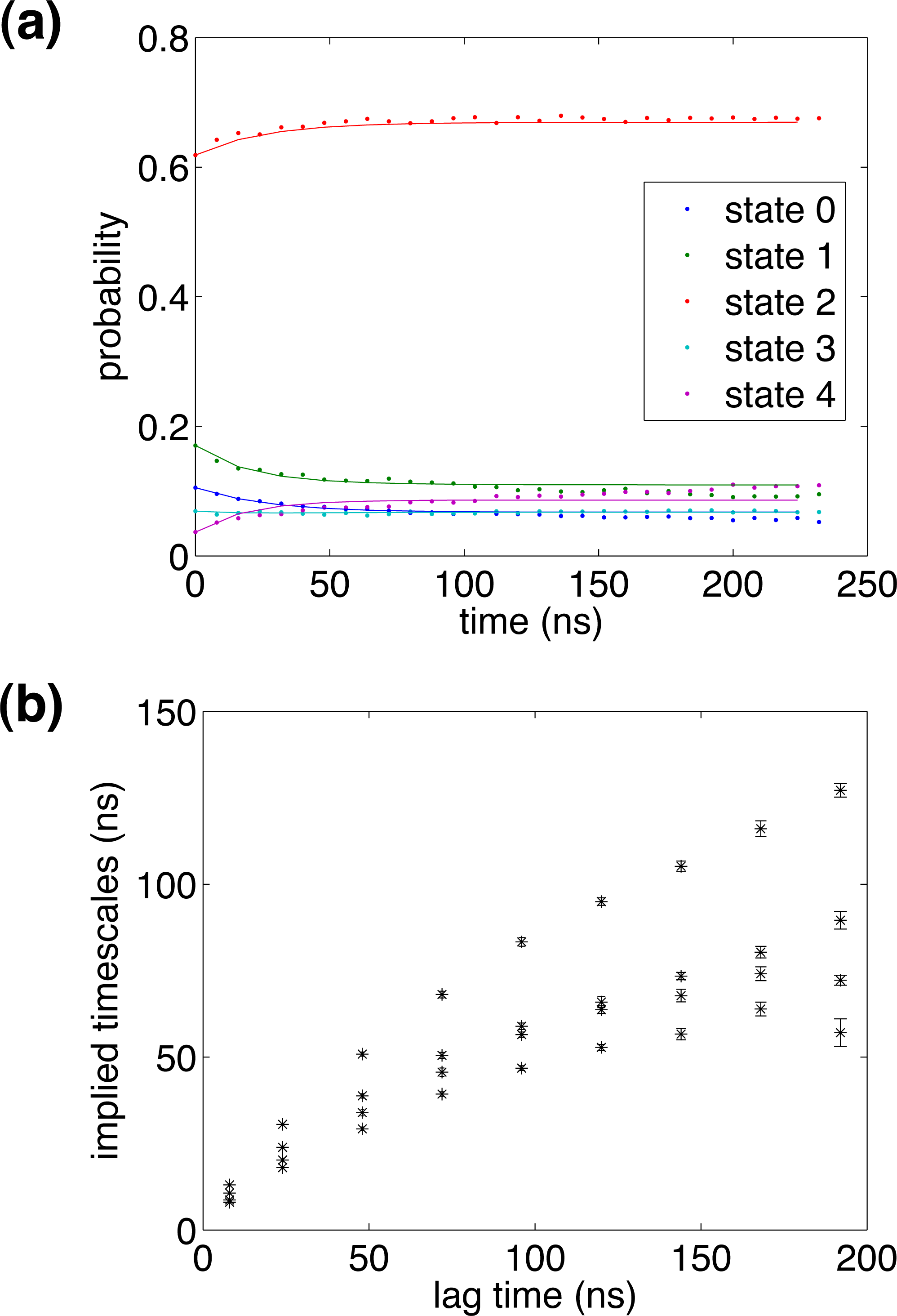

Supplement: Figure S2. — (a) A MSM built using a lag time of τ=240 ns reproduces the time evolution of macrostate populations, (b) As the lag time used to build the MSM increases, so do the implied timescales (shown with error bars from a simple bootstrapping procedure). Regardless of lag time, the implied timescales do not show a pronounced separation of timescales. [file ijms-10-01013f13.tif]

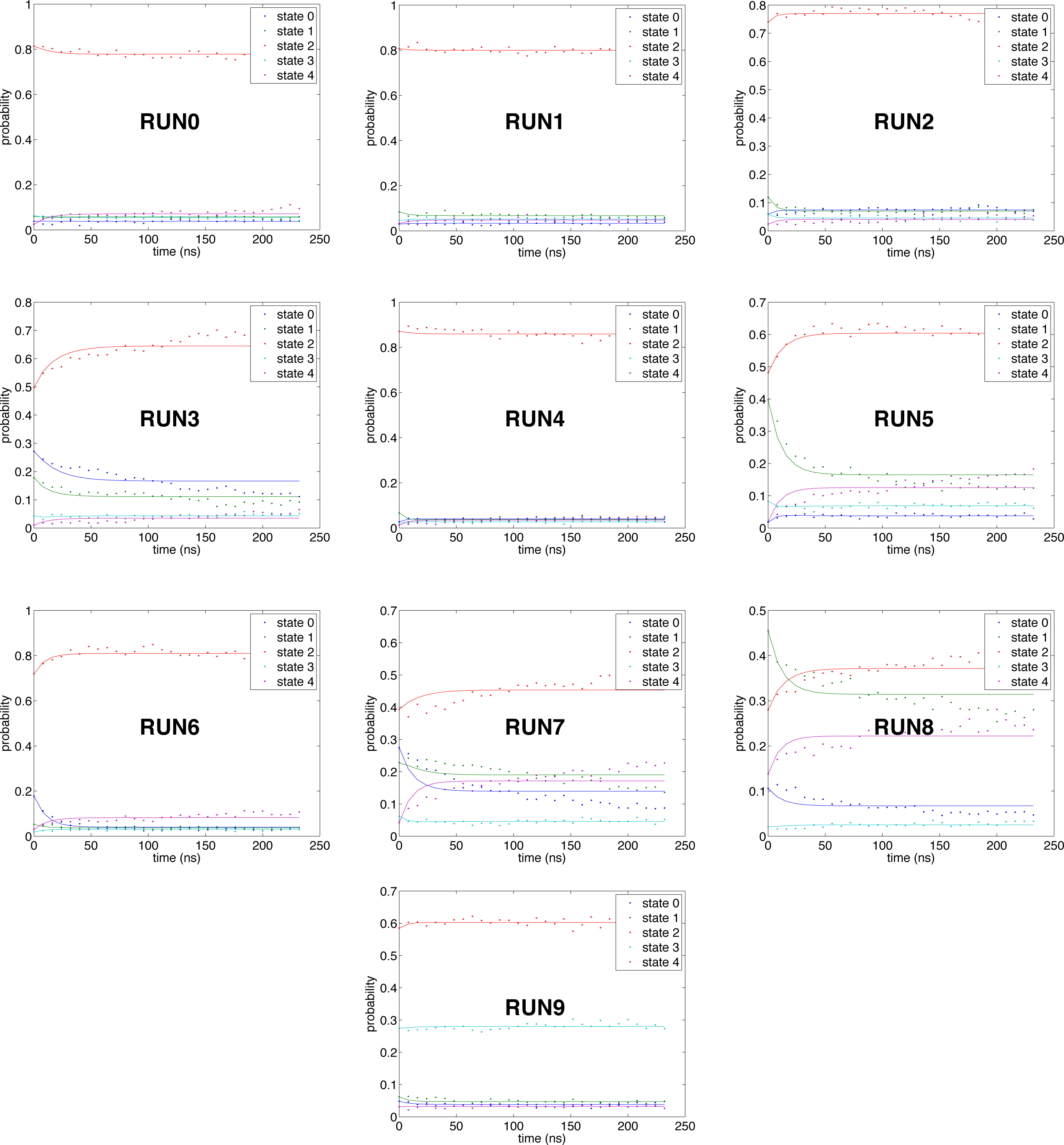

Supplement: Figure S3. — Markov State Models built from trajectory data for each starting conformation, each constructed using a short lag time of τ=8 ns. MSM predictions of the macrostate population time evolution is shown as the solid line; the actual macrostate populations over time are shown as dots. [file ijms-10-01013f14.tif]

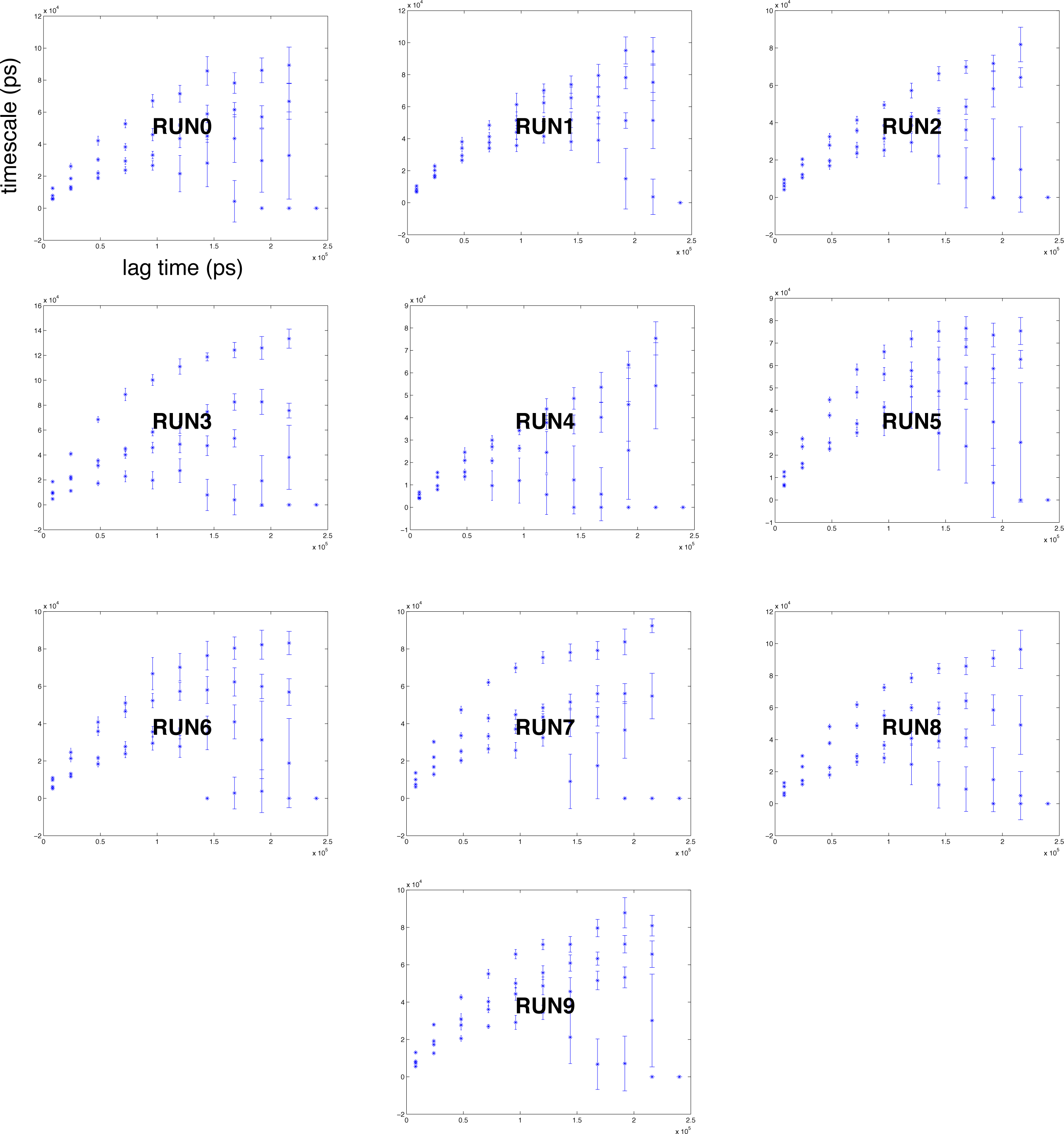

Supplement: Figure S4. — Implied timescales as a function of lag time for MSMs constructed for each starting conformation. Error estimates (bars) for timescales at each lag time were derived from a bootstrapping procedure. [file ijms-10-01013f15.tif]

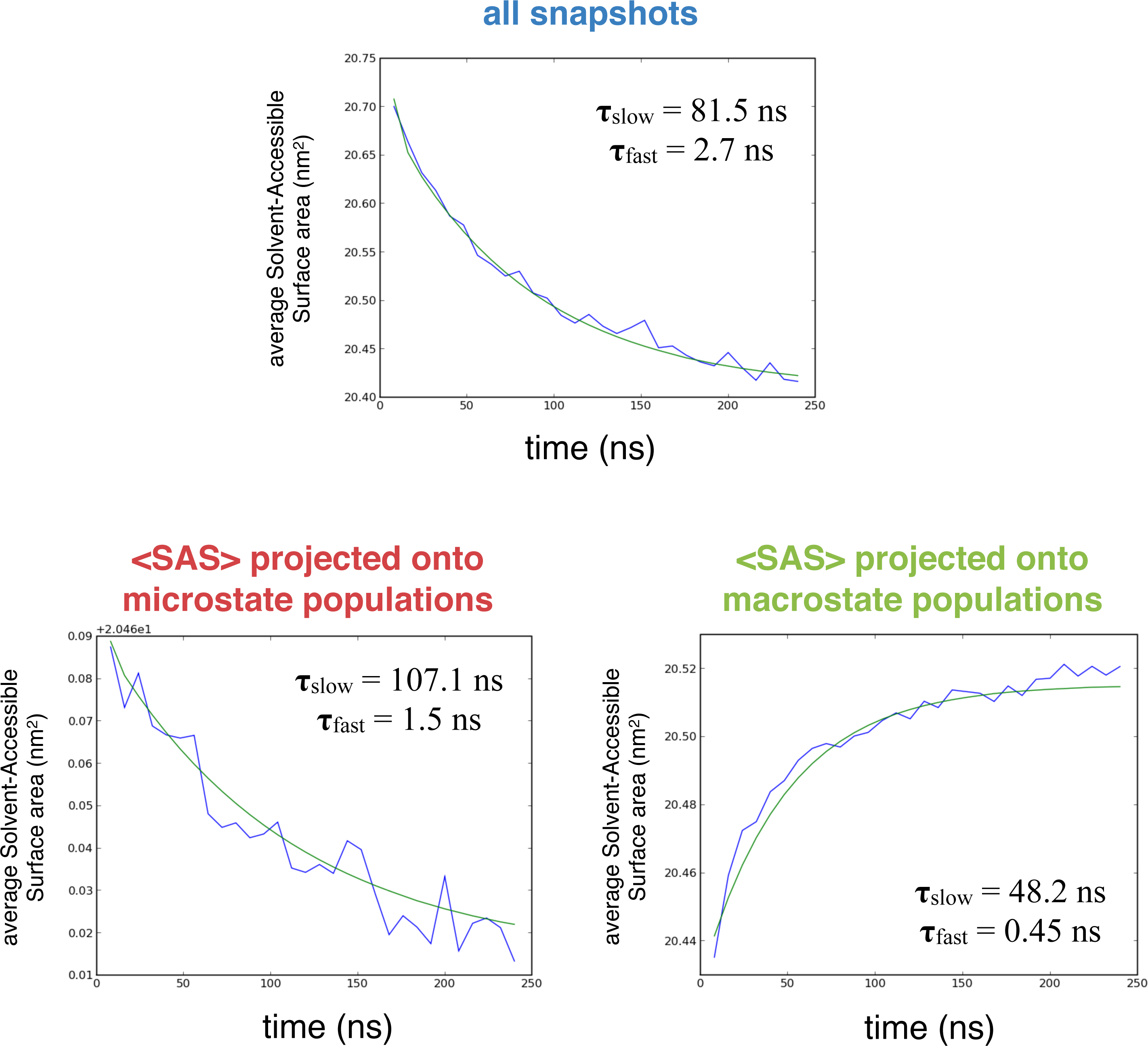

Supplement: Figure S5. — Bi-exponential fits of the average C=O solvent-accessible surface area (SAS) over time computed from simulation snapshot data (blue), compared to the average SAS of each microstate projected onto the 4000 microstate populations over time (red), and average SAS of each macrostate projected onto 5 macrostate populations over time (green). Despite the differential effects produced by averaging over microstates and macrostates, the relaxation time scales are similar. [file ijms-10-01013f16.tif]
